# Association Between Adequacy and Moderation of Quality of Diet with Metabolic Syndrome Parameters Among Iranian Health Workers Based on the Baseline Data of Employees Health Cohort Study

**DOI:** 10.34172/aim.33193

**Published:** 2025-04-01

**Authors:** Mohammad Hossein Sharifi, Elahe Mansouriyekta, Seyed Jalil Masoumi, Alireza Mirahmadizadeh, Zahra Rostami Ghotbabadi

**Affiliations:** ^1^Research Center for Traditional Medicine and History of Medicine, Shiraz University of Medical Sciences, Shiraz, Iran; ^2^Non-Communicable Diseases Research Center, Shiraz University of Medical Sciences, Shiraz, Iran; ^3^Nutrition Research Center, School of Nutrition and Food Sciences, Shiraz University of Medical Science, Shiraz, Iran; ^4^Gastroenterohepatology Research Center, Shiraz University of Medical Sciences, Shiraz, Iran; ^5^Employee Cohort Study, Shiraz University of Medical Sciences, Shiraz, Iran; ^6^School of Medicine, Shiraz University of Medical Sciences, Shiraz, Iran

**Keywords:** Adequacy, Healthy eating index, Metabolic syndrome, Moderation

## Abstract

**Background:**

A healthy diet is essential for managing metabolic syndrome (MetS), but moderation and dietary adequacy remain ambiguous.

**Methods:**

Data from the recruiting phase of the Shiraz University of Medical Sciences Employees Health Cohort Study (SUMS EHCS) were utilized to conduct this cross-sectional analysis. A validated 168-item semi-quantitative food frequency questionnaire (FFQ) was used to collect dietary data in the Persian cohort. In the current study, the healthy eating index (HEI-2015) includes two components, namely adequacy and moderation which were used to evaluate the quality of the diet.

**Results:**

The study included 3380 health workers, with a mean age of 41.81±7 years and 55.2% female. Among them, 22.3% met the ATP III criteria for MetS. The mean total HEI, adequacy, and moderation scores were 63.89±9.53, 41.03±5.88, and 20.13±4.90, respectively. Adjusted model analysis showed no significant correlation between diet adequacy and MetS or its components, but found a significant association between diet moderation and MetS (OR: 1.03 [1.008–1.05]), abdominal obesity (OR: 1.02 [1.003–1.04]), elevated serum triglycerides (TGs) (OR: 1.02 [1–1.03]), and elevated fasting blood sugar (FBS) (OR: 1.03 [1.005–1.05]).

**Conclusion:**

This study found that there was a significant correlation between diet moderation and abdominal obesity, elevated serum TGs, elevated FBS, and MetS. Future studies on the topic are recommended.

## Introduction

 Metabolic syndrome (MetS) is a collection of metabolic risk factors and lifestyle modifications. Notably, a healthy diet and physical activity constitute the main therapeutic approach to MetS management.^1^ A healthy diet is defined in a variety of ways, including Dietary Guidelines for Americans (DGA), Recommended Dietary Allowance, Healthy Eating Index (HEI) 2015, Mediterranean diet, and others.^2,3^ The majority of people, especially the youth, fail to meet dietary requirements, or eat a low-quality diet.^4^ Evidence indicates that improving nutrition quality can lower the burden of chronic diseases such as obesity, diabetes, cardiovascular disease, and cancer.^5,6^ The HEI 2015 covers a subset of dietary quality, adequacy, and moderation and adherence to this pattern could potentially prevent chronic diseases.^7^ Although some studies have been published on the relationship between diet quality and MetS, the association between MetS parameters and moderation and adequacy of diet has yet to be confirmed.^2^

 The association between MetS and diet quality has been inconsistently reported in published articles.^2,8,9^ The Konikowska et al study in 2015 found that MetS subjects had considerably lower total HEI-2015 scores than those in the control group, although a prior study found that adherence to HEI-2015 had no significant link with the prevalence of MetS and its components in the Iranian population.^8,9^ It is worth noting that the relationship between MetS and diet quality subscales such as adequacy and moderation was not assessed.

 While nutritional adequacy is described as consuming enough nutrients to meet the human body’s requirements for optimal health, it is also a crucial component of the diet quality assessment in HEI 2015.^7^ There are different definitions and scales for adequacy of diet. In most scientific literature, nutrient adequacy is the level of intake of an essential nutrient in relation to the nutrient requirement for adequate health, which is expressed as the percentage of recommended dietary allowance. However, based on the HEI 2015, adequacy of diet consists of nine components including total fruits, whole fruits, total vegetables, greens and beans, whole grains, dairies, total protein foods, seafood and plant proteins, and fatty acids.^7^ The study by Sharifi *et al.* in 2022 indicated that waist circumference, low-density lipoprotein (LDL) cholesterol, and total cholesterol were influenced by adequacy of diet quality in cardiovascular diseases.^10^ Studies have been published on the relationship between nutritional adequacy and MetS, but these studies have used different definitions of nutritional adequacy; to the best of our knowledge, the current study is the first study to address the relationship between adequacy of diet based on the HEI 2015 and MetS parameters.^11,12^ Therefore, the exploration of detailed information on the relationship between adequacy of diet and MetS parameter could help health workers to provide suitable dietary advice and personalized messages for patients with MetS.

 Moderation, generally understood to mean not eating too much or too little, is the primary element considered when evaluating the quality of a diet in the HEI 2015.^7^ The basis of the moderation in HEI 2015 is emphasizing to limit the intakes of sodium, added sugars, saturated fat, and refined grains.^7^ Previous studies indicated that waist circumference, LDL, and high-density lipoprotein (HDL) cholesterol were influenced by moderation of diet quality in cardiovascular diseases.^10^ To the best of our knowledge, the current study is the first study regarding the relationship between moderation of diet based on the HEI 2015 and MetS parameter.

 The global scientific evidence showed an increasing trend of the prevalence of MetS worldwide; to improve MetS, enhanced diet quality is emphasized. Dietary advice based on adequacy and moderation could be useful in practice to provide personalized messages to improve MetS parameters. To the best of our knowledge, the current study is the first study regarding the relationship between adequacy and moderation of diet based on the HEI 2015 and MetS parameter.

## Materials and Methods

###  Study Design 

 We conducted a cross-sectional study to investigate the relationship between the HEI including adequacy and moderation and MetS parameters among 3,380 health workers aged 21 to 65 years. Data were used from the recruiting phase of the Shiraz University of Medical Sciences Employees Health Cohort Study (SUMS EHCS) which lasted from 2018 to 2022.

###  Cohort Description 

 SUMS EHCS is a branch of the PERSIAN cohort study launched by the Ministry of Health and Medical Education of Iran in 2014. The objective of this cohort is to identify the most prevalent non-communicable diseases in Iranian ethnic groups and to examine effective preventive measures.^13^ A trained team verified the voluntary participation of individuals meeting the inclusion criteria as per the PERSIAN protocol. Participants received a briefing about the cohort, and informed written consent was obtained. They represented various occupational groups, including administrative, clinical therapeutic, public service, technical and maintenance, laboratory staff, and security. Individuals with physical or mental disabilities who could not complete the registration process, as well as pregnant women, were excluded. Each participant was assigned a unique digital code for identification, ensuring data confidentiality. Central and local teams ensured compliance with the PERSIAN Cohort protocol through quality assurance and control measures, which included daily data entry monitoring, periodic reviews for missing information, and surprise inspections at cohort sites.

###  Assessments

 Participants completed a general questionnaire covering demographic factors, occupational status, past medical history (with a focus on non-communicable diseases), and personal habits, including smoking history, opium and hookah use, as well as alcohol consumption. Additionally, physical activity levels were assessed using the International Physical Activity Questionnaire (IPAQ). Anthropometric measurements, including height, weight, and waist circumference, were taken in the morning while participants were in a fasting state. Blood pressure readings, including systolic and diastolic measurements, were recorded using the InBody BPBIO 320 digital device in a sitting state. After a 12-hour fasting period, blood samples were collected using Vacutainers (KANG JIAN, China, (K2EDTA-9 mL), Golden Vac, China, (K2EDTA-3 mL), AYSET TUBE, Turkey, (Clot Activator-6 mL)).

###  Calculation of Healthy Eating Index, Adequacy and Moderation Score

 A validated and reliable 168-item semi-quantitative food frequency questionnaire (FFQ) was utilized to gather dietary data in the Persian cohort.^13^ Expert dieticians conducted face-to-face interviews to assess the frequency of consumption for each food item. Participants reported their consumption over the past year on a monthly, weekly, or daily basis. After data collection, all findings were converted to a daily intake scale. Finally, the Nutritionist IV software, modified for Iranian foods (version 7.0; N-Squared Computing, Salem, OR, USA), was employed to analyze the macro- and micro-nutrient content of the food items.^14^ In the current study, the HEI-2015 that includes the two componentsofadequacy (total fruits, whole fruits, total vegetables, greens and beans, whole grains, dairies, total protein foods, seafood and plant proteins, and fatty acids) and moderation (refined grains, sodium, added sugars, and saturated fats) was used to evaluate the quality of the diet. Adequacy has a maximum total score of 60 and moderation has a maximum total score of 40. For both components, higher scores suggest better diet quality.

###  Metabolic Syndrome 

 This study used the NCEP ATP III criteria to define MetS, and it was diagnosed when three or more of the following five criteria were met: Abdominal obesity (waist circumference > 88 cm in women and > 102 cm in men), low serum HDL-C ( < 40 mg/dL in males; < 50 mg/dL in females), triglycerides (TGs) ( ≥ 150 mg/dL) or drug treatment, elevated fasting blood sugar (FBS) ( ≥ 100 mg/dL) or drug treatment, and elevated blood pressure (systolic ≥ 130 mm Hg and/or diastolic ≥ 85 mm Hg) or drug treatment.^15^

###  Statistical Analysis

 Analyses were performed using the SPSS software (version 25) and significance was considered < 0.05. Categorized variables were reported as number (percentages), while continuous variables were reported as mean (standard deviation). We used one-way analysis of variance (ANOVA) to compare the mean of adequacy and moderation in the general characteristics of participants. We also investigated the relationship between the total adequacy and moderation score and MetS and its components in crude and adjusted models using binary logistic regression tests. The covariates included sex, marital status, age, education, physical activity, body mass index (BMI), employment status, and smoking and alcohol consumption. The adjusted model included covariates with *P* value > 0.2 in the crude model.

## Results

 A total of 3380 health workers with a mean ± SD age of 41.81 ± 7 years, 55.2% female and 44.8% male were included in the study. The mean ± SD for daily calorie intake was 2174.63 ± 730.36. Also, 22.3% of the participants met the ATP III criteria for MetS. The most prevalent component of MetS was abdominal obesity (Table 1). The mean ± SD values for adequacy and moderation were 41.03 ± 5.88 and 20.13 ± 4.90, respectively.

**Table 1:** General Characteristics and Biochemistry Parameters of Participants by Mean ± SD of Adequacy, Moderation and HEI in Baseline Data of EHWC Cohort Study of SUMS

**Characteristics Variable**		**Number (%)**	**Adequacy **		**Moderation **		**HEI**	
			**Mean±SD**	***P*** **Value**^a^	**Mean±SD**	***P*** **Value**^a^	**Mean±SD**	***P*** **Value**^a^
Sex	Female	1866 (55.2)	42.3 ± 5.6	**<0.001**	19.9 ± 5.2	**0.001**	65.67 ± 9.77	**<0.001**
	Male	1514 (44.8)	39.5 ± 5.8		20.4 ± 4.5		61.69 ± 8.75	
Marital status	Single	482 (14.3)	41.4 ± 5.7	0.12	20.6 ± 5.2	**0.006**	64.74 ± 9.78	**0.03**
	Married	2737 (81)	41 ± 5.9		20.1 ± 4.8		63.82 ± 9.47	
	Divorced	161 (4.8)	40.3 ± 5.9		19.2 ± 5.7		62.58 ± 9.82	
Age	≤ 40	1553 (45.9)	40.9 ± 5.8	0.16	20.1 ± 4.8	0.86	63.02 ± 9.78	**<0.001**
	> 40	1827 (54.1)	41.2 ± 5.9		20.1 ± 4.9		64.63 ± 9.62	
Education	Illiterate or elementary	137 (4.1)	38.1 ± 6.9	**<0.001**	19.1 ± 4.5	**0.002**	59.21 ± 10.16	**<0.001**
	Middle	138 (4.1)	37.7 ± 6.7		19.5 ± 4.8		58.50 ± 9.68	
	High school and diploma	613 (18.1)	39.6 ± 5.9		19.8 ± 4.8		61.47 ± 9.37	
	College	2492 (73.7)	41.7 ± 5.6		20.3 ± 5		65.04 ± 9.24	
Physical activity (MET^b^ min/wk)	Low ( < 600)	1555 (46)	41.7 ± 5.8	**<0.001**	20.3 ± 4.9	**0.01**	65.05 ± 9.36	**<0.001**
	Moderate (600–2999)	638 (18.9)	41.4 ± 5.5		20.4 ± 4.8		64.75 ± 9.29	
	High ( ≥ 3000)	1187 (35.1)	40 ± 6.1		19.8 ± 4.9		61.91 ± 9.58	
BMI (kg/m^2^)	< 18.5	33 (1)	40.8 ± 6	0.32	20.2 ± 4.8	0.77	62.24 ± 8.68	0.36
	18.5-24.9	1115 (33.5)	40.9 ± 5.6		19.5 ± 3.8		63.59 ± 9.63	
	25-29.9	1556 (46.8)	41.2 ± 5.7		20.1 ± 5		64.14 ± 9.40	
	≥ 30	620 (18.7)	41 ± 6		20.2 ± 5		63.84 ± 9.82	
Employment status	Official	1583 (46.9)	41.9 ± 5.9	**<0.001**	20.1 ± 4.9	0.74	65.39 ± 9.22	**<0.001**
	Contracts	200 (5.9)	41.4 ± 5.8		20 ± 5		64.06 ± 9.52	
	Probationary or corporate	1587 (47.1)	40.2 ± 6.1		20.2 ± 4.9		62.42 ± 9.57	
Smoking	Never and former	3164 (93.7)	41.1 ± 5.9	0.056	20.1 ± 4.9	0.45	63.97 ± 9.58	0.057
	Current	213 (6.3)	40.3 ± 5.7		20.4 ± 4.9		62.69 ± 8087	
Alcohol use	No	3233 (95.7)	40.7 ± 5.9	0.46	19.7 ± 4.9	0.29	63.95 ± 9.56	0.07
	Yes	144 (4.3)	41.1 ± 5.9		20.2 ± 4.9		62.50 ± 8.98	
MetS^c^	No	2617 (77.7)	41 ± 5.9	0.15	20 ± 4.9	**0.004**	63.62 ± 9.49	**0.003**
	Yes	753 (22.3)	41.3 ± 5.8		20.6 ± 4.9		64.78 ± 9.66	
MetS components	Abdominal obesity							
	No	1738 (51.6)	40.5 ± 5.9	**<0.001**	20.1 ± 4.7	0.71	63.01 ± 9.25	**<0.001**
	Yes	1632 (48.4)	41.6 ± 5.8		20.2 ± 5.1		64.81 ± 9.75	
	Elevated blood pressure^d^							
	No	2822 (83.7)	41.07 ± 5.9	0.28	20 ± 4.9	**0.03**	63.90 ± 9.61	0.81
	Yes	548 (16.3)	40.8 ± 5.6		20.5 ± 4.7		63.79 ± 9.17	
	Elevated serum TGs^d^							
	No	2138 (63.4)	41.2 ± 5.9	**0.02**	19.9 ± 4.9	**0.006**	64.02 ± 9.62	0.28
	Yes	1232 (36.6)	40.7 ± 5.8		20.4 ± 4.8		63.65 ± 6.38	
	Low serum HDL-C							
	No	1974 (58.6)	40.8 ± 5.9	**0.03**	20.2 ± 4.9	0.62	63.72 ± 9.52	0.24
	Yes	1396 (41.4)	41.3 ± 5.8		20.1 ± 5		64.11 ± 9.56	
	Elevated FBS^d^							
	No	2800 (83.1)	41 ± 5.8	0.92	20 ± 4.9	**0.006**	63.69 ± 9.50	**0.01**
	Yes	570 (16.9)	41 ± 6		20.6 ± 5		64.80 ± 9.69	

Data are reported as Mean ± standard deviation (SD) and n (%). ^a^ANOVA test was used; Statistical significance was set at the level of *P* < 0.05. ^b^Metabolic equivalents. ^c^According to the ATP III criteria, MetS was defined as the presence of three or more of the following components: Abdominal obesity (waist circumference > 88 cm in women and > 102 cm in men), low serum HDL-C ( < 40 mg/dL in males; < 50 mg/dL in females), TGs ( ≥ 150 mg/dL) or drug treatment, elevated FBS ( ≥ 100 mg/dL) or drug treatment, and elevated blood pressure (systolic ≥ 130 mm Hg and/or diastolic ≥ 85 mm Hg) or drug treatment; ^d^Drug treatment. Bolded values indicate that the *P* value is < 0.05.

 The general characteristics and biochemistry parameters of the participants by mean ± SD of adequacy, moderation and HEI are provided in Table 1. The mean of adequacy was significantly higher among women, health workers with office jobs, those with university education, and those who had low physical activity. Male, singles, and university-educated health workers as well as those who engaged in low physical activity had a significantly higher mean of moderation (Table 1). The mean of HEI was higher in females than males, and among single health workers compared to married and divorced ones. It was also higher for those over 40 years of age than for those under 40, and for health workers with official employment compared to those with probationary or corporate positions. Additionally, health workers with higher education had a higher mean of HEI than those with lower education. Those with lower physical activity levels had a higher HEI than those with higher physical activity. Furthermore, health workers who consumed alcohol had a higher HEI than those who did not (Table 1).

Table 2 shows the HEI-2015 components scores. The mean ± SD for the total HEI score was 63.89 ± 9.53.

**Table 2:** HEI-2015 Components Scores in Baseline Data of EHWC Cohort Study of SUMS

**HEI-2015 components**		**Range of Score**	**Mean Score±SD**
Adequacy	Total fruits	0 - 5	4.48 ± 0.94
	Whole fruits	0 - 5	4.81 ± 0.49
	Total vegetables Green and beans	0 - 5 0 - 5	4.84 ± 0.5 2.64 ± 1.20
	Whole grains	0 - 10	6.50 ± 3.41
	Dairies	0 - 10	4.81 ± 2.14
	Total protein foods	0 - 5	4.42 ± 0.80
	Seafood and plant proteins	0 - 5	4.55 ± 0.80
	Fatty acids	0 - 10	3.86 ± 3.16
Moderation	Refined grains	0 - 10	2.38 ± 3.31
	Sodium	0 - 10	5.88 ± 3.48
	Added sugars	0 - 10	5.29 ± 3.61
	Saturated fats	0 - 10	9.29 ± 1.48
Total score		0 - 100	63.89 ± 9.53

 Examining the crude association between the adequacy and MetS and its components showed a statistically significant association between adequacy and elevated serum TGs, abdominal obesity, and low serum HDL-C (Table 1). However, abdominal obesity (OR: 1.03; 95% CI: 1.02, 1.05) remained significant in the adjusted model (Table 3).

**Table 3:** Adjusted Odds Ratios with 95% Confidence Interval for the Association betweenHEI (Adequacy & Moderation)and Metabolic Syndrome and its Components in Baseline Characteristics EHWC Cohort Study of SUMS

**Dependent Variable**	**Adequacy**		**Moderation**		**HEI**	
	**OR (95% CI)**	***P*** ** Value**	**OR (95% CI)**	***P*** ** Value**	**OR (95% CI)**	***P*** ** Value**
MetS	1.01 (0.99–1.03)^a^	0.21	1.03 (1.008–1.05)^a^	**0.005**	1.011 (1.001–1.021)	**0.02**
MetS components						
Abdominal obesity	1.034 (1.018–1.051)^b^	**<0.001**	1.01 (0.99–1.028)^b^	0.23	1.022 (1.013–1.032)	**<0.001**
Elevated blood pressure	1.014 (0.99–1.03)^c^	0.12	1.019 (0.99–1.04)^c^	0.07	1.01 (0.99–1.021)	0.08
Elevated serum TGs	1.003 (0.99–1.017)^d^	0.64	1.015 (0.99–1.03)^d^	0.06	1.005 (0.99–1.013)	0.25
Low serum HDL-C	0.99 (0.98–1.01)^e^	0.72	1.004 (0.98–1.018)^e^	0.63	0.99 (0.99–1.006)	0.61
Elevated FBS	1.008 (0.99–1.03)^f^	0.34	1.03 (1.005–1.05)^f^	**0.01**	1.013 (1.003–1.024)	**0.01**

Logistic regression analysis was used to determine the association. Statistical significance was set at the level of *P*< 0.05. Bolded values indicate that the *P *value is < 0.05. The covariates included sex, marital status, age, education, physical activity, BMI, employment status, and smoking and alcohol consumption. The adjusted model included covariates with *P *value > 0.2 in the crude model. ^a^Adjusted for marital status, age, education, physical activity, BMI, employment status and calorie intake. ^b^Adjusted for marital status, age, education, physical activity, BMI, employment status and calorie intake. ^c^Adjusted for sex, marital status, age, education, BMI, employment status and calorie intake. ^d^Adjusted for sex, marital status, age, education, physical activity, BMI, employment status, alcohol consumption and calorie intake. ^e^Adjusted for sex, age, education, physical activity, BMI, alcohol consumption and calorie intake. ^f^Adjusted for sex, marital status, age, education, physical activity, BMI, employment status and alcohol consumption.

 Also, in the crude model, there was a statistically significant association between moderation and MetS, elevated blood pressure, elevated serum TGs, and elevated FBS (Table 1). The adjusted model showed that MetS (OR: 1.03; 95% CI: 1.008, 1.05) and elevated FBS (OR: 1.03; 95% CI: 1.005, 1.05), remained significant (Table 3).

 Investigating the crude association between the HEI and MetS and its components showed a statistically significant association between HEI and MetS, abdominal obesity and elevated FBS (Table 1). In the adjusted model, MetS (OR: 1.011; 95% CI: 1.004, 1.021), abdominal obesity (OR: 1.022; 95% CI: 1.013, 1.032), and elevated FBS (OR: 1.013; 95% CI: 1.003, 1.024) remained significant (Table 3).

## Discussion

 The primary management approach for MetS is dietary lifestyle modifications. The roles of the two healthy-eating indices of moderation and adequacy in MetS have yet to be determined. Our adjusted model analysis revealed that there were no significant correlations between dietary adequacy and MetS and its components. However, we found a significant link between moderation of diet and MetS, abdominal obesity, high blood TGs, and elevated FBS.

 In developed countries, the prevalence of MetS has risen up to 20%–25% in the adult population, and its prevalence continues to grow over the years.^2^ MetS increases the chance of T2DM onset and major cardiovascular events by two and five folds, respectively. Other chronic diseases such as cancer, neurodegenerative diseases, non-alcoholic fatty liver disease, lipid and circulatory disorders, atherosclerosis, and all disorders leading to mortality are increased.^2^ The current study discovered that 22.3% of health workers had MetS, but a meta-analysis of 69 studies revealed that 30.4% of the Iranian population had MetS.^16^ This means that MetS is less common among healthcare workers compared to the general population.

 Some experts emphasize the correlation between the adequacy of diet and possible chronic factors together with obesity, hypertension, diabetes, and MetS parameters. Adequacy means that the food should meet the nutritional requirements of each person, based on their age, gender, body size, and level of physical activity.^17^ Adequacy is also one of the main parts of the updated HEI 2015 that includes intake of total fruits, whole fruits, total vegetables, greens and beans, whole grains, dairies, total protein foods, seafood and plant proteins, and fatty acids.^7^ The current study found that the mean of adequacy was significantly higher among women, health workers with office jobs and university education, and those who had low physical activity. However, the adjusted model analysis did not show a correlation between adequacy and metabolic parameters syndrome including abdominal obesity, elevated blood pressure, elevated triglyceride levels, low HDL, and elevated FBS. This result is inconsistent with previous studies which found a significant positive correlation between waist circumference and nutrient adequacy.^18,19^ One explanation for this is the different methods used to assess nutritional adequacy. The most common method of measuring nutritional adequacy is administering a food frequency questionnaire and evaluating the intake of macronutrients and micronutrients according to individual nutritional requirements.^19^ However, the current study assessed the adequacy of diet based on HEI-2015 with MetS. It is notable that the assessment of the adequacy of diet based on HEI-2015 with MetS parameters is limited.

 The moderation of eating patterns is linked to chronic disease risk factors like arthrosclerosis, diabetes, and MetS and it is one of the key components of the diet quality and HEI -2015 index.^7^ In general, moderation refers to eating neither excessively nor insufficiently. Furthermore, moderation is a key component of the updated HEI 2015 assessment for refined grains, salt, added sugars, and saturated fats.^7^ In other words, the moderation components in HEI 2015 indicate food groups and dietary nutrients for which intake limits are recommended. The adjusted model analysis of the current study demonstrated that there was a significant connection between moderation and MetS, abdominal obesity, high serum TGs, and elevated FBS. In agreement with this finding, a recent study found that waist circumference and body mass index were associated with the moderation of diet quality.^10^ According to de Oliveira Otto et al in 2015, moderation in eating does not contribute to better metabolic health, but rather diversity in food is associated with abdominal obesity.^20^ Compared to the findings of the current study, one possible explanation for this outcome is the difference in approach and tools of assessing the moderation of food quality.^20^ Most published research appears to evaluate HEI in terms of health outcomes such as mortality and cardiovascular diseases with little attention placed on HEI subgroups (adequacy and moderation) and health metabolic outcomes.^21^ Saraf-Bank et al in 2017 found that low food quality based on the HEI-2010 can be linked to MetS components such as systolic and diastolic blood pressure, TG, and BMI. However, in this study, the researchers did not assess the association of HEI with MetS components.^22^

 One of the strengths of the current study was its large sample size. However, because this study obtained data only from health workers, the findings cannot be generalized to all individuals. Health workers likely have greater health literacy and access to healthcare. Therefore, our estimates for MetS and HEI may underestimate rates in the broader population. Another limitation of this study is the potential recall bias in dietary reporting, as FFQs depend on participants’ memory, which can be inaccurate. Additionally, social desirability bias may lead participants to report healthier eating habits than they actually follow. In addition, another problem was lack of similar findings from other studies with which to compare.

## Conclusion

 Experts tend to focus on intervention aimed at healthy dietary patterns for managing MetS rather than limiting any one nutrient, and there is rising scientific evidence supporting the importance of health food quality and patterns in non-communicable diseases. The data demonstrated that lifestyle changes can play a significant role in managing MetS, and further information in this field can be beneficial. This study found that while there was no significant correlation between an adequate diet and MetS or any of its components, there was a significant correlation between diet moderation and abdominal obesity, elevated serum TGs, elevated FBS, and MetS. As there are few published articles regarding the relationship between diet moderation and MetS components, future studies on this topic are recommended.
